# Strength Characteristics of a Smooth HDPE Geomembrane/Nonwoven Geotextile Interface Based on a Novel Ring Shear Apparatus

**DOI:** 10.3390/polym15112497

**Published:** 2023-05-29

**Authors:** Zhanlei Liu, Jianyong Shi, Hai Lin, Yuchen Zhang

**Affiliations:** 1 Key Laboratory of Ministry of Education for Geomechanics and Embankment Engineering, Hohai University, Nanjing 210024, China; liu_zhanlei@hhu.edu.cn (Z.L.); zhangyuchen@hhu.edu.cn (Y.Z.); 2 School of Infrastructure Engineering, Nanchang University, Nanchang 330031, China; linhai@ncu.edu.cn

**Keywords:** smooth geomembrane, nonwoven geotextile, peak strength, residual strength, high normal stress, submerged conditions

## Abstract

This paper aims to investigate the interfacial strength characteristics, particularly the residual strength, of a high-density polyethylene smooth geomembrane (GMB-S)/nonwoven geotextile (NW GTX) interface using a novel ring shear apparatus under high normal stresses and two specimen conditions. A total of eight normal stresses (from 50 kPa to 2308 kPa) and two specimen conditions (dry and submerged at ambient temperature) are considered in this study. The reliability of using the novel ring shear apparatus to study the strength characteristics of the GMB-S/NW GTX interface was demonstrated by conducting a series of direct shear experiments with a maximum shear displacement of 40 mm and ring shear experiments with a shear displacement of 10 m. The peak strength, post-peak strength development, and residual strength determination method of the GMB-S/NW GTX interface are explained. Three exponential equations suitable for characterizing the relationship between the post-peak friction angle and the residual friction angle of the GMB-S/NW GTX interface are established. This relationship can be used with the relevant apparatus (i.e., an apparatus with deficiencies in executing large shear displacement) in determining the residual friction angle of the high-density polyethylene smooth geomembrane/nonwoven geotextile interface.

## 1. Introduction

Geotextiles are commonly used as protective layers in combination with geomembranes to form composite liners to help prevent puncture damage caused by high normal stress and angular soil particles [1,2,3,4,5,6,7,8]. A composite liner system containing a smooth geomembrane (GMB-S) and a nonwoven geotextile (NW GTX) was laid at the bottom of the site during the construction of the Kettleman Hills landfill [9]. The GMB-S/NW GTX interface was one of the interfaces where instability and failure of the landfill occurred in 1988 [10,11]. In applications involving drainage filtration, separation, protection, and reinforcement, a composite system is typically employed by combining GMB-S and NW GTX rather than using standalone units [7,8,12]. The interfacial shear strength of the liner system incorporating GMB-S and NW GTX is crucial to the stability of overlying structures supported by the system.

Published experimental data on the smooth geomembrane/geotextile interfacial strength indicate that the maximum normal stress that could be applied using a ring shear apparatus was 480 kPa [13]. However, the normal stress acting on the bottom lining system of the majority of municipal solid waste landfills has exceeded 480 kPa [14,15,16,17,18,19,20,21,22,23,24,25,26]. This normal stress has even exceeded 2 MPa in some leachate ponds [27,28]. Therefore, the experimental apparatus currently available for studying the strength characteristics of the GMB-S/NW GTX interface is clearly insufficient in terms of the loading capacity of normal stress. In addition, the currently available shear experimental results for smooth geomembrane/geotextile interfaces reveal linearity in all peak friction angles, large displacement friction angles, and even residual friction angles. However, the trends of these friction angles under high normal stress (greater than 480 kPa) have rarely been reported. Landfill caps are also typical applications of liner systems that include geomembranes and geotextiles, which permanently sustain shear stress when applied in slopes. Normally, an area load of 50 kPa is applied in accordance with the typical upper limit in slopes of landfill caps. Compared to the bottom lining system of a landfill site, measuring its friction behavior solely through short-term shear box experiments is not sufficient, as aging, creep, and stress cracking are important factors affecting its stability, and long-term shear experiments also must be considered [29]. Shear displacement that is detrimental or damaging can cause post-peak strength mobilization during landfill construction and operation, leading to overestimation of the shear strength of the liner system [30]. This overestimation can easily result in an unstable landfill and require substantial remediation costs [9,10,11,13,31,32,33]. The differences in tensile strain between the geotextile and geomembrane laid on the slope of a landfill may result in a relative interfacial shear displacement up to 1.3 m [34] or even larger [1]. This suggests that although there are currently no signs of instability or destruction in the landfill, there has been a certain amount of internal shear displacement within the liner system. However, there are still few published studies on the shear displacement of GMB-S/NW GTX interfacial experiments that can exceed 1.3 m, regardless of the normal stress and specimen conditions. It has not been verified whether the interfacial strength of the liner system composed of GMB-S and NW GTX has reached its residual strength within a shear displacement of 1.3 m. Therefore, further research is required to investigate the strength characteristics of the GMB-S/NW GTX interface with variation in shear displacement interfacial strength. The use of a direct shear apparatus is common due to its simple and straightforward mechanical principle and the ability to maintain a constant shear direction along a predefined plane [35]. Many researchers have used a direct shear apparatus to study the strength properties of the GMB-S/NW GTX interface [2,3,4,5,6,7,10,36,37,38,39,40,41]. However, the direct shear apparatus does have certain limitations in terms of large shear displacement [35]. In recent years, both traditional and improved ring shear apparatuses have been developed for studying interfacial strength (particularly in the field of residual strength) due to their advantages of arbitrary shear displacement and constant shear area. Significant achievements have been made in the study of the interfacial strength properties of geosynthetic materials using the ring shear apparatus [13,42,43,44,45,46,47].

The aim of this study is to achieve four objectives. The first is to verify the feasibility of using the ring shear apparatus to study the strength characteristics of the GMB-S/NW GTX interface. The second is to evaluate the influence of high normal stress and specimen conditions (dry and submerged) on the peak interfacial strength and post-peak large displacement strength of the GMB-S/NW GTX interface. The third is to propose a scientific method for determining the residual strength of the GMB-S/NW GTX interface. The fourth is to establish a functional relationship between the residual strength and post-peak large displacement strength of the GMB-S/NW GTX interface, to compensate for the deficiency of the direct shear apparatus in terms of large shear displacement and to simplify the functional relationship into a ratio of peak strength to residual strength for more convenient use by engineers in practical applications. 

## 2. Experimental Work

### 2.1. Testing Apparatus

**Figure 1:**
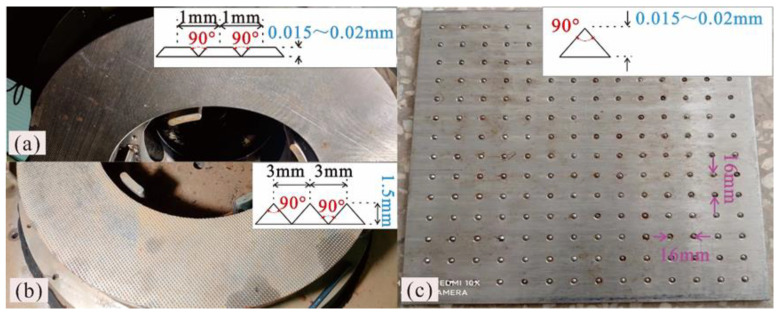
(**a**) Nail plate panel for clamping NW GTX in the ring shear apparatus; (**b**) nail plate panel for clamping GMB-S in the ring shear apparatus; (**c**) nail plate panel for clamping NW GTX and GMB-S in the direct shear apparatus.

**Figure A1:**
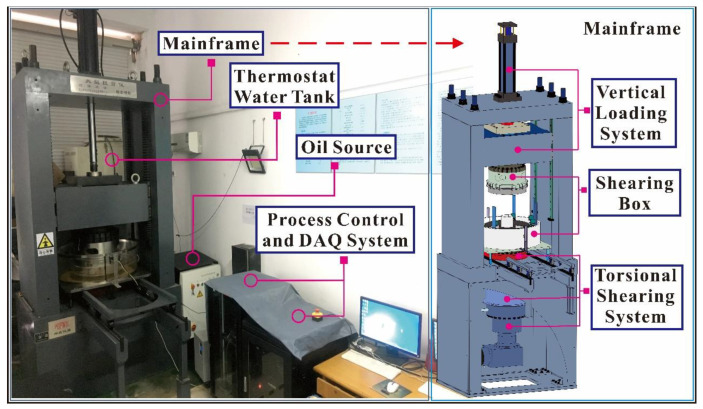
Picture and main structure of the novel ring shear apparatus.

This study employs two types of apparatus, namely, a novel ring shear apparatus (Shear testing machine, Hohai University, Nanjing, China) [35] and a large direct shear apparatus (Shear testing machine, Nanchang University, Nanchang, China) [48]. The design objective of the novel ring shear apparatus is to study the interfacial strength behavior of the liner system at any arbitrary displacement [35], and its structural schematic is shown in Figure A1 in Appendix A. The specimen is a ring with a 300 mm inner diameter and a 500 mm outer diameter. The vertical loading system is a hydraulic servo drive system based on computer instructions, and it can apply a normal stress of up to 2.39 MPa to the specimen and measure its vertical deformation. The torsional shearing system is a servo drive system also based on computer instructions, which can apply a desired shear stress of up to 2.59 MPa to the specimen and measure its torsional shearing displacement. The multifunctional shearing box was simplified for use in the ring shear experiment of the GMB-S/NW GTX interface, and its primary function is to clamp the specimen and provide a submerged environment during the experiment. Figure 1a,b show the specially designed clamps for the GMB-S and NW GTX specimens. The surface of the nail plate used for clamping the GMB-S is processed with uniformly distributed pyramid-shaped nails, with a height of 1.5 mm and a spacing of 3 mm between the nail tips, as shown in Figure 1b. The surface of the nail plate used for clamping the NW GTX was first processed into a pyramid with the same shape as the GMB-S nail board, but the height of the nails and the spacing between the nail tips were 0.5 mm and 1 mm, respectively. Then, the top of the pyramid was processed using a grinder, with a remaining height of 0.015 mm to 0.02 mm, as shown in Figure 1a. This was performed to ensure that the nail plate had sufficient friction force against the NW GTX and to completely prevent the nail spike from piercing the NW GTX during the experimental process, which would affect the accuracy of the experimental results. The large direct shear apparatus developed by Lin et al. [48] was selected to test the peak strength characteristics of the GMB-S/NW GTX interface. The experimental results were compared with those of the novel ring shear experiment to characterize the effect of the shear stress state on the interfacial shear strength. The goal was to verify the feasibility of the novel ring shear apparatus in studying the strength properties of the GMB-S/NW GTX interface. The shear box of this apparatus is a pair of rectangular plates, both 300 mm in length and width. Many uniformly distributed adjustable height nails were installed on each rectangular plate, which were used to clamp the specimen. Its function is similar to that of the nail plate panel of the novel ring shear apparatus, as shown in Figure 1c. The normal stress is powered by the hydraulic cylinder based on the computer instructions, and the maximum normal stress is 2.8 MPa. The shear stress is powered by a stepper motor that drives the ball screw, and the maximum shear displacement is 40 mm. The values of stresses and displacements during the experiment were monitored by a force sensor and a linear displacement sensor, respectively. The shear stress and normal stress in this experiment had to be corrected, as the shear area gradually decreased during the shear experiment.

### 2.2. Description of Materials

The focus of this study is the interface between the GMB-S and NW GTX materials. The GMB-S is a high-density polyethylene (HDPE) geomembrane with a nominal thickness of 2.0 mm per ASTM D5199 [49]. It is manufactured using the blown film process. The NW GTX is a polyester geotextile made of staple fibers and needle-punched, with a mass per unit area of 800 g/m^2^ per ASTM D5261 [50].

### 2.3. Test Procedures

**Figure 2:**
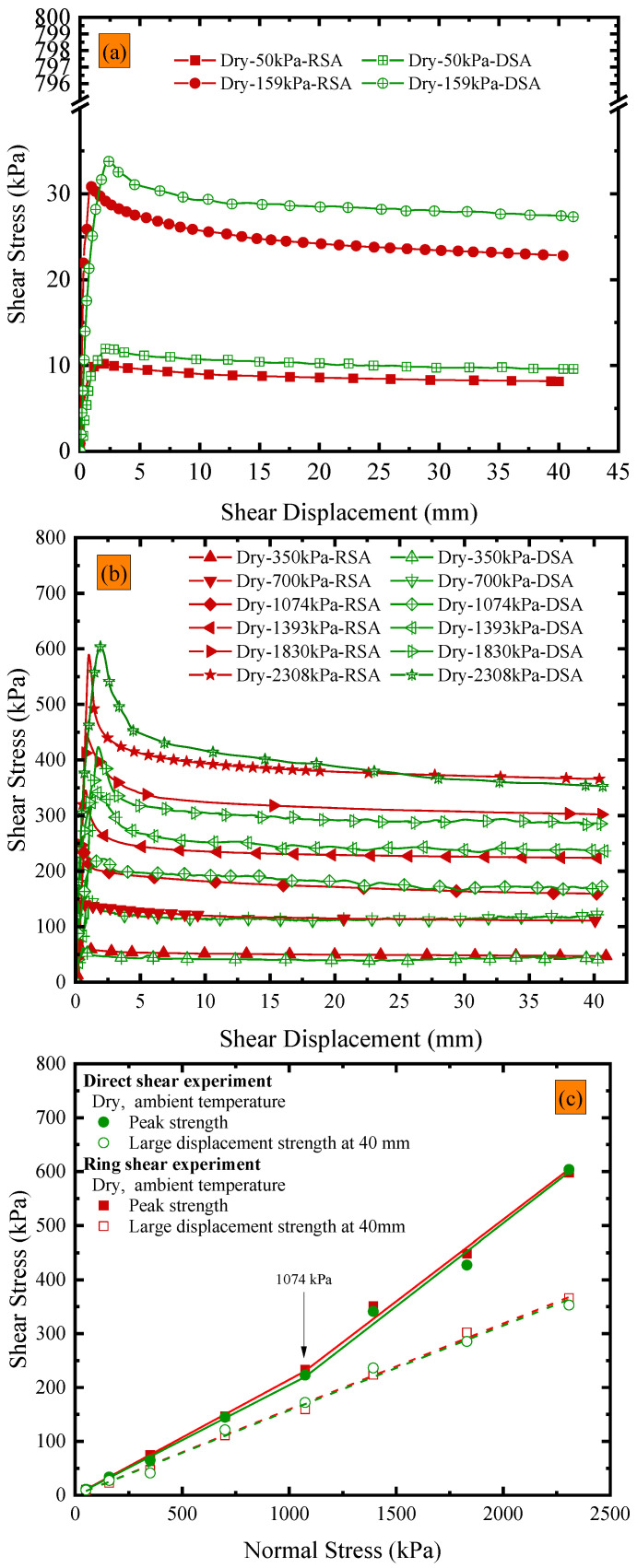
Comparation of data from direct shear and ring shear experiments under dry conditions at ambient temperature: (**a**,**b**) variations in shear stress vs. shear displacement; (**c**) the peak strength envelopes.

This experiment utilizes the displacement control mode which strictly adheres to ASTM 5321 [51] and 6243 [52]. Therefore, there were no significant differences in the basic operation of the direct shear and ring shear experiments. The specific operation processes are as follows:

- Clamp selection and installation: Nail plates, as shown in Figure 1, were utilized to clamp the specimens in this experiment. Specifically, Figure 1a,b show how the nail plates were applied to the ring shear apparatus to clamp the GMB-S and NW GTX, respectively. Figure 1c displays the nail plate used for clamping the GMB-S and NW GTX in the direct shear apparatus. The distribution and size of the nails on the nail plate are depicted in Figure 2.
- Specimen preparation and installation: The NW GTX and GMB-S specimens were initially coarsely cut using the trapezoidal sampling method and then finely cut using a specimen preparation device. The direct shear apparatus utilized a rectangular specimen of 300 × 300 mm^2^, while the ring shear apparatus utilized an annular specimen of Φ300 mm/Φ500 mm. In the next step, the NW GTX and GMB-S specimens were installed on their corresponding nail plates. For the ring shear experiment, the combined specimens had to remain on the same axis. It is important to note that the GMB-S specimens should be wiped with a hand towel before experiments, as recommended by previous studies [53,54].
- Setting specimen conditions: The specimens were subjected to two different conditions: dry and submerged. For the submerged conditions, the thermostatted water tank shown in Figure 1 was utilized to provide circulating water. Although leachate from landfills may have a long-term impact on the interfacial strength of GMB-S/NW GTX [55], it is not the focus of this study. Tap water was used as the medium for the submerged condition in this research.
- Loading normal stress: The vertical loading system was initiated by the control system, and the normal stress was applied gradually to the combined specimen until it reached the target value. The normal stresses utilized in this experiment were 50 kPa, 159 kPa, 700 kPa, 1393 kPa, 1830 kPa, and 2308 kPa. Once the normal stress reached the target value, it was maintained constant for no less than 40 min to ensure the combined specimen thickness remained steady.
- Loading shear stress: The control system initiated the displacement control mode of the shear system by command, with a shear displacement rate of 5 mm/min in this experiment. This value conforms to ASTM 5321 [51] and 6243 [52]. The target shear displacements for the direct shear experiment and the ring shear experiment were 40 mm and 10 m, respectively. Case studies of instability in landfills caused by the low shear strength of the liner system have shown that the Kettleman landfill underwent a maximum horizontal displacement of 10.668 m after instability in 1988 [32,56]. However, horizontal displacements in other unstable landfills are much smaller than 10.668 m [57]. Therefore, the maximum shear displacement of 10 m selected in this experiment was acceptable.
- Stopping and unloading the specimen: The experiment ended and the specimen was unloaded when the shear displacement reached the set value. All specimens used in the experiment were numbered.

The details of the experimental program are summarized in Table 1.

## 3. Results and Discussion

### 3.1. Feasibility of the Ring Shear Apparatus for the GMB-S/NW GTX Interface

Shear experiments under dry conditions at an ambient temperature were conducted on the GMB-S/NW GTX interface using a large direct shear apparatus and a novel ring shear apparatus, and the variations in shear stress with shear displacement are shown in Figure 2a,b, respectively. As mentioned in Section 2.3, the shear displacement in the ring shear experiment was 10 m, and thus the data from the ring shear experiment in Figure 2a,b were extracted from its complete dataset. The purpose of this selection was to facilitate a comparative analysis with the data obtained from the direct shear experiments. Figure 2a,b demonstrate that the shear displacement required for the peak shear strength of the GMB-S/NW GTX interface in the ring shear experiment was in the range of 0.61 to 1.99 mm when calculated based on the middle diameter of the circular specimen, and in the range of 0.76 to 2.49 mm when calculated based on the outer diameter of the circular specimen. The shear displacement required for the peak shear strength of the GMB-S/NW GTX interface in the direct shear experiment was mostly within the range of 2.12 to 3.06 mm. It can be inferred that the shear displacement required for the peak shear strength of the GMB-S/NW GTX interface in the ring shear experiment was slightly less than that in the direct shear experiment. There were concave marks on the NW GTX specimen, which was punctured (but not pierced) by nails during the direct shear experiment, and obvious tearing along the shearing direction. In addition, the material between two adjacent concave marks (perpendicular to the shearing direction) showed obvious displacement along the shearing direction, which was more obvious in the middle closer to the adjacent concave marks. In contrast, although there were concave marks on the NW GTX specimen, which was punctured by nails during the ring shear experiment, tearing and displacement were negligible. As shown in Figure 1a,c, the distance between nails on the clamping nail plate used in the direct shear experiment was 16 mm, which is much larger than the 1 mm between nails used in the ring shear experiment. The denser the nails on the clamping nail plate, the better the stability of clamping the NW GTX specimen. Therefore, the difference in the shear displacement required to obtain peak strength using the two apparatuses was caused by the different specifications of the nail plate. The two shear displacement intervals mentioned above, which are required to drive the peak strength, did not show a significant difference from the historical results of the GMB-S/NW GTX interfacial shear experiments [2,36,39,41].

The peak strength and post-peak strength (at 40 mm) data based on the direct shear experiment and ring shear experiment are shown in Figure 2c. The maximum deviation of peak strength was 15.2%, which occurred at a normal stress of 350 kPa. The maximum deviation of post-peak strength at 40 mm was 16%, which occurred at a normal stress of 159 kPa. The deviation of post-peak strength at 40 mm under a normal stress of 50 kPa was 14.9%, and under a normal stress of 350 kPa, it was 14.2%. The peak strength and post-peak strength (at 40 mm) under the remaining normal stresses deviate by less than 10%. The reasons for the abovementioned deviation include not only the differences in the shear stress state between the direct shear and the ring shear apparatus, but also the nonuniformity of the experimental material caused by manufacturing processes, which cannot be ignored. 

As shown in Figure 2c, the peak strength envelopes of the GMB-S/NW GTX interface for both direct shear and ring shear experiments exhibit obvious nonlinear characteristics within the range of normal stresses from 50 kPa to 2308 kPa. Considering various factors, a bilinear regression equation with a cross-over point of 1074 kPa is suitable for describing the peak strength envelope of the GMB-S/NW GTX interface. After linear fitting in the normal stress range of 50 kPa to 1074 kPa, the peak friction angles obtained from direct shear and ring shear experiments were 11.55° and 12.11°, respectively, with a difference of 0.56°. The peak friction angles were 17.11° and 16.87°, respectively, with a difference of 0.24°, in the range of normal stresses from 1074 kPa to 2308 kPa. Compared with the peak strength envelope, the post-peak friction angle at a shear displacement of 40 mm showed linear characteristics within the range of normal stresses from 50 kPa to 2308 kPa. The post-peak friction angles at a shear displacement of 40 mm for the direct shear and ring shear experiments were 8.95° and 9.06°, respectively, resulting in a difference of 0.11° in the post-peak friction angles. As described above, the differences in peak and post-peak friction angles were both less than 1°, which is within an acceptable range for engineering applications. Based on the experimental results and analysis, it can be concluded that the use of the ring shear apparatus is a reliable method for studying the strength characteristics of the GMB-S/NW GTX interface. The measured friction angles obtained in this experiment are compiled in Table 2.

### 3.2. Influence of High Normal Stress on Peak Strength and Residual Strength

Historical research on the strength characteristics of the GMB-S/NW GTX interface has shown maximum normal stress values of 480 kPa [13] and maximum shear displacement of 1150 mm [58]. Furthermore, both the peak and post-peak strength envelopes show linear behavior under dry conditions at ambient temperature. However, the peak strength envelope of the GMB-S/NW GTX interface exhibits clear bilinear behavior within the normal stress range from 50 kPa to 2308 kPa in this study, with a cross-over point at a normal stress of 1074 kPa. This finding suggests that the friction coefficient of the GMB-S/NW GTX interface varies with increasing normal stress, which is in agreement with the observation made by Kim and Frost (2011) that the coefficient between geosynthetic materials tends to stabilize or increase with increasing normal stress [3]. Hence, the weight of the overlying material must be considered as a crucial factor in projects that use GMB-S and NW GTX as a bottom lining system. To ensure a reliable design, the appropriate interface peak friction coefficient should be selected based on the normal stress magnitude, as opposed to using a constant friction coefficient.

**Figure 3:**
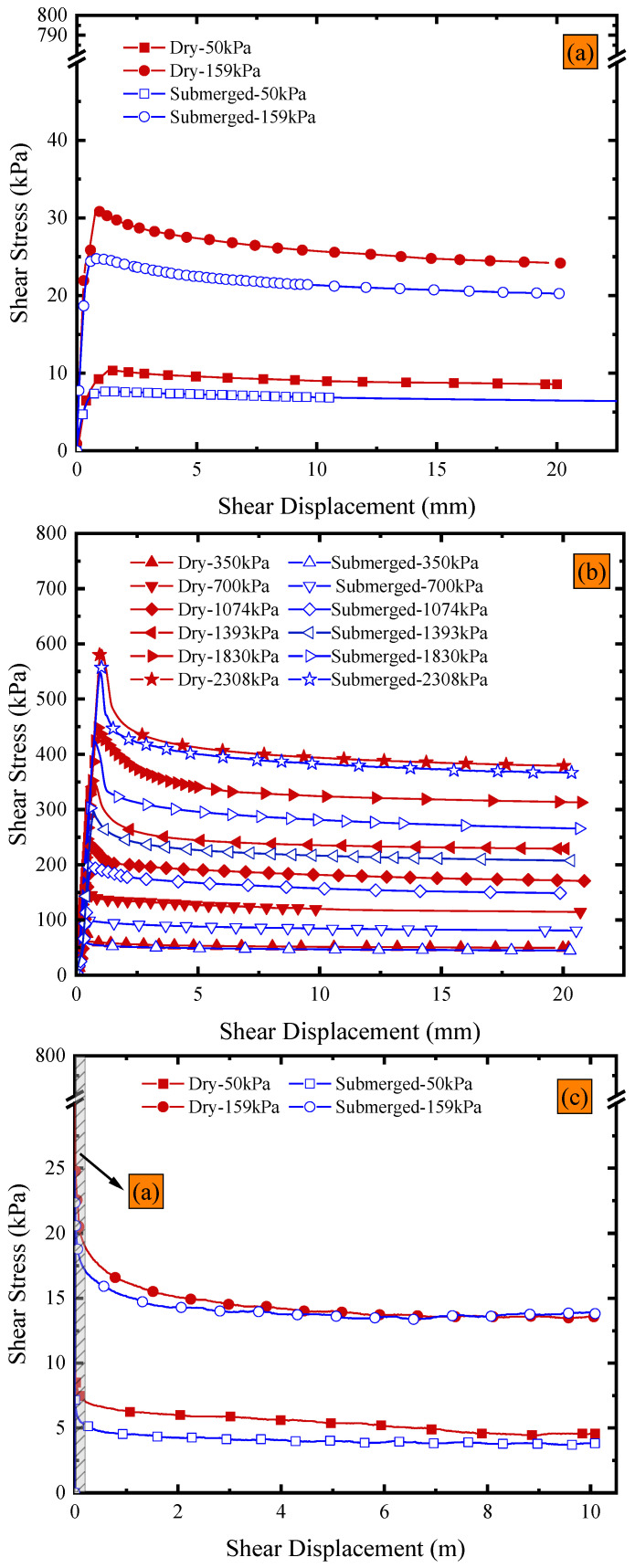

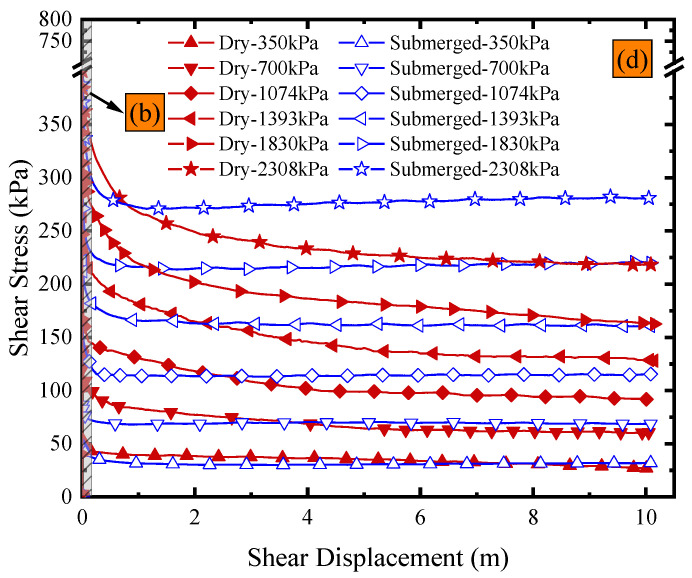
Variations in shear stress vs. shear displacement based on the ring shear apparatus under dry and submerged conditions of ambient temperature: (**a**,**b**) the peak shear stress part; (**c**,**d**) the shear displacement of 10 m.

**Figure 4:**
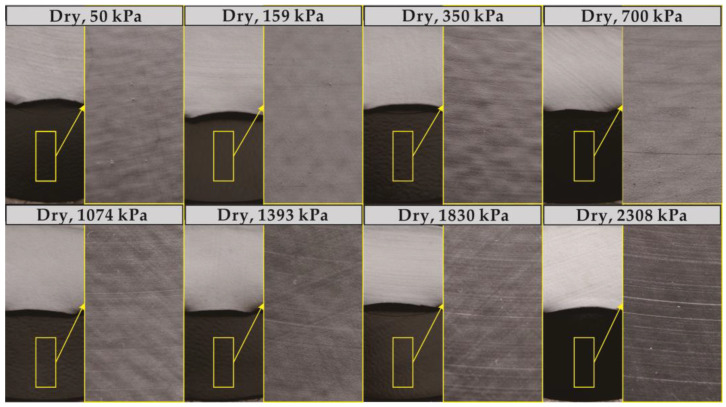
Images of the GMB-S and NW GTX specimens after the ring shear experiment conducted under dry ambient temperature conditions.

**Figure 5:**
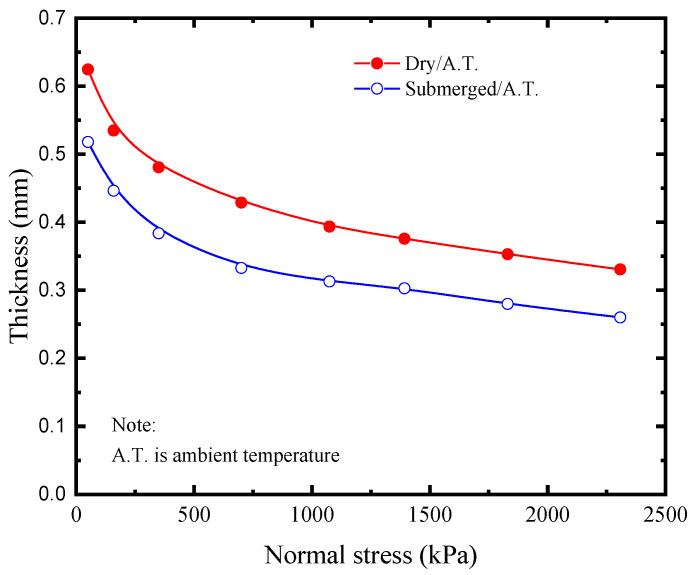
Variations in NW GTX thickness with normal stress after the experiment.

The development trend of the GMB-S/NW GTX interfacial strength with increasing shear displacement is illustrated in Figure 3a–d (plotted using solid symbols), considering dry conditions at ambient temperature and the range of normal stress from 50 kPa to 2308 kPa. The rate of decrease in post-peak shear stress gradually decreases as the shear displacement increases. However, the post-peak shear stress still exhibits a continuous decreasing trend within the shear displacement of 10 m. The images of the GMB-S and NW GTX specimens after the ring shear experiment conducted under dry conditions at ambient temperature are shown in Figure 4. The GMB-S specimens had almost no wear after shearing for 10 m under low normal stresses of 50 kPa, 159 kPa, and 350 kPa, and only exhibited slight circular scratches at a normal stress of 700 kPa. As the normal stress increased, these circular scratches became more pronounced. The variation in the thickness of the NW GTX specimen with respect to normal stress after the experiment is presented in Figure 5. The thickness of the NW GTX specimen after the experiment, under a normal stress of 2308 kPa, was 0.33 mm, which was significantly greater than the height of the nail on the NW GTX specimen clamping plate, measuring 0.02 mm. Therefore, the circular scratches on the surface of the GMB-S specimen in Figure 4 were not caused by the nail on the clamping plate penetrating the NW GTX specimen, but rather by plowing the NW GTX specimen on the surface of the GMB-S specimen under high normal stress.

**Figure 6:**
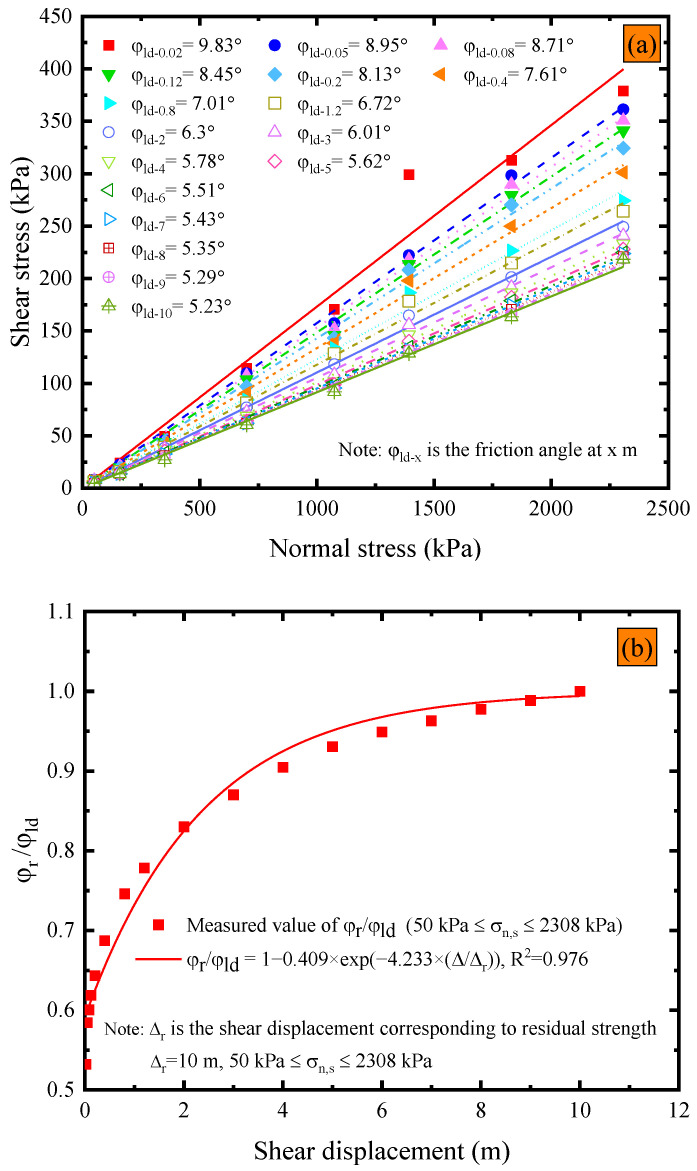
Dry conditions at ambient temperature: (**a**) variations in post-peak strength envelope with shear displacement; (**b**) variations in the ratio of residual friction angle to post-peak large displacement strength friction angle vs. shear displacement.

ASTM D5321 [51] and D6243 [52] state that the shear force can be considered to have reached a steady state once it has peaked and shows no significant increase or decrease for a shear displacement of 12.7 mm after reaching the peak. However, these specifications do not provide any reference regarding the extent to which the shear stress does not increase or decrease significantly. Figure 6a illustrates the impact of shear displacement on the post-peak strength envelope under dry conditions at ambient temperature. The post-peak interface friction angles within the shear displacement range from 0.02 m to 10 m were linear. Additionally, the results also reveal that the post-peak strength friction angle gradually decreases as the shear displacement increases, and the rate of decrease appears to decrease with a further increase in shear displacement. The maximum shear displacement in the field of research on the strength of the interface between smooth geomembranes and nonwoven geotextiles is 1.15 m. The shear displacement closest to this value, as recorded in Figure 6a, is 1.2 m, with a corresponding peak post-peak friction angle of 6.72°. However, the post-peak friction angle at a shear displacement of 10 m is 5.23°, which is 22.2% less than the post-peak friction angle observed at 1.2 m. Accurate determination of interfacial strength parameters is crucial for ensuring long-term stability in engineering applications, as overestimating the interfacial strength can potentially result in safety hazards. The maximum shear displacement used in this experiment was 10 m. However, it should be acknowledged that it is unknown whether the post-peak shear strength of the GMB-S/NW GTX interface tends to stabilize after exceeding a shear displacement of 10 m. Nevertheless, a shear failure of 10 m has already resulted in landfills being in a state of failure, as confirmed by failure cases in solid waste landfills [57]. Therefore, it may be reasonable to assume that the shear stress at a shear displacement of 10 m can be used as the residual strength for subsequent analysis of the GMB-S/NW GTX interface under dry conditions at ambient temperature.

Figure 6b depicts the relationship between the ratio of residual strength friction angle (φ_r_) to post-peak strength friction angle (φ_ld_) and shear displacement. It is worth noting that φ_r_ refers to the post-peak strength friction angle at a shear displacement of 10 m. The development of post-peak strength with shear displacement was described using an exponential function with two parameters, as proposed by [59,60]. The equation is presented in (1):

(1) $$\varphi_{r}=\left(1-A\times \exp\left(B\times \left(\frac{\Delta_{\mathrm{ld}}}{\Delta_{r}}\right)\right)\right)\times \varphi_{\mathrm{ld}}$$

where the Δ_ld_ is the shear displacement at any position after the shear stress exceeds the peak strength in the GMB-S/NW GTX interface shear experiment, Δ_r_ is the shear displacement required to measure the residual strength friction angle, and the units of Δ_ld_ and Δ_r_ are m. By substituting the 17 data points presented in Figure 6a,b into Equation (1), the coefficients A, B, and Δ_r_ are determined to be 0.41, −4.23, and 10, respectively. Therefore, an exponential equation, as shown in Equation (2), was utilized to describe the relationship between residual strength and post-peak displacement intensity for the GMB-S/NW GTX interface under dry conditions and at ambient temperature. The corresponding coefficients are tabulated in Table 3.

(2) $$\varphi_{r}=\left(1-0.41\times \exp\left(-4.23\times \left(\frac{\Delta_{\mathrm{ld}}}{10}\right)\right)\right)\times \varphi_{\mathrm{ld}}$$

### 3.3. Influence of Submerged Conditions on Peak Strength and Residual Strength

**Figure 7:**
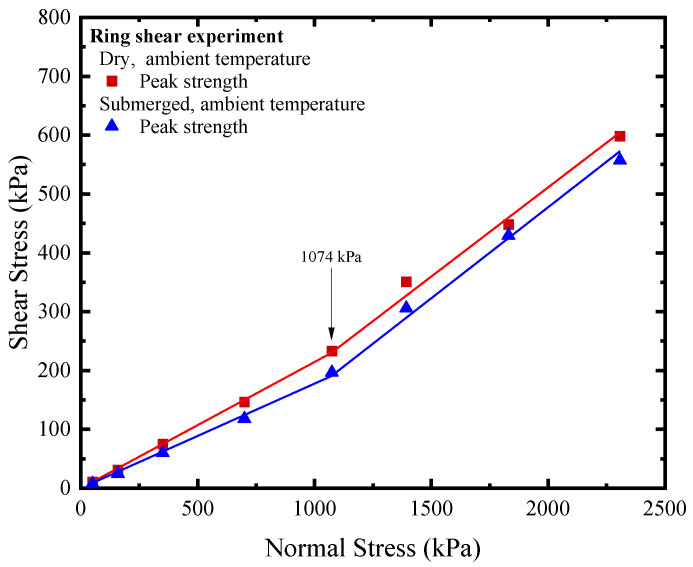
Peak strength envelopes of ring shear experiments on the GMB-S/NW GTX interface under dry and submerged conditions at ambient temperature.

The study on the impact of submerged conditions on the peak and residual strength of the GMB-S/NW GTX interface is discussed in the context of the ring shear experiment. The variations in the peak strength part of the shear stress vs. shear displacement under submerged conditions at ambient temperature are shown in Figure 3a,b (plotted using open symbols). The development trend is consistent with that under dry conditions, where the shear stress rapidly reaches its peak strength with a small shear displacement, followed by a gradual decrease with increasing shear displacement. The peak strength of the GMB-S/NW GTX interface exhibited significant nonlinear characteristics with respect to normal stress under submerged conditions, as shown in Figure 7. Considering various factors such as engineering applications, the peak strength envelope was represented using a bilinear approach. Multiple fitting tests showed that the optimal cross-over point was at 1074 kPa, which was the same as the position under dry conditions. This indicates that the specimen state (dry and submerged) did not affect the development of peak strength at the GMB-S/NW GTX interface in the normal stress range from 50 kPa to 2308 kPa. Specifically, the peak friction angle was 10.08° in the normal stress range from 50 kPa to 1074 kPa, and 17.09° in the range of 1074 kPa to 2308 kPa. The corresponding friction angle data can be found in Table 2. A comparison showed that the peak friction angle of the GMB-S/NW GTX interface under submerged conditions was 2.03° smaller than that under dry conditions within the normal stress range from 50 kPa to 1074 kPa at ambient temperature. This finding is in line with earlier studies [2,10,40,41]. However, the peak friction angle of the GMB-S/NW GTX interface under submerged conditions is nearly identical to that under dry conditions within the high normal stress range of 1074 kPa to 2308 kPa, as shown in Table 2. Nevertheless, this does not imply that the strength characteristics of the GMB-S/NW GTX interface under dry and submerged conditions are the same within the high normal stress range. The peak strength at the cross-over points of 1074 kPa is different, with peak strengths of 233.32 kPa and 196.5 kPa under dry and submerged conditions, respectively. This indicates that submerged conditions have minimal impact on the peak friction angle of the GMB-S/NW GTX interface within the high normal stress range from 1074 kPa to 2308 kPa but do have a certain weakening effect on its peak strength.

The variations in shear stress with a shear displacement of 10 m under submerged conditions at ambient temperature are illustrated in Figure 3c,d (plotted using open symbols). The post-peak shear stress behavior of the GMB-S/NW GTX interface shows significant differences between submerged and dry conditions as shear displacement progresses, manifesting in three specific aspects. First, the peak strength of the GMB-S/NW GTX interface under submerged conditions was less than that under dry conditions. However, the rate of weakening of the post-peak strength of the GMB-S/NW GTX interface under dry conditions was larger than that under submerged conditions with increases in shear displacement. Specifically, the post-peak strength of the GMB-S/NW GTX interface under dry conditions gradually became less than that under submerged conditions as the shear displacement increased. Moreover, the shear displacement corresponding to the intersection of the shear stress curves for these two specimen conditions also decreased gradually with an increase in normal stress, as shown in Figure 3c,d. Second, as described in Section 3.2, the post-peak strength of the GMB-S/NW GTX interface under dry conditions consistently decreased within the range of 10 m of shear displacement. In comparison, the post-peak strength trend of the GMB-S/NW GTX interface was significantly different under submerged conditions. Specifically, the post-peak strength of the GMB-S/NW GTX interface under submerged conditions gradually stabilized with increasing shear displacement under normal stresses other than 1830 kPa and 2308 kPa, as shown in Figure 3c,d (plotted using open symbols). Third, the post-shear strength of the GMB-S/NW GTX interface initially decreased to a minimum value and then gradually increased under high normal stresses. As shown in Figure 3d, the post-peak strength first decreased to its minimum shear stress of 213.72 kPa and 270.76 kPa under normal stresses of 1830 kPa and 2308 kPa, respectively. Then, the post-peak shear strength slowly increased again with the development of shear displacement. Compared to the minimum shear stress, the post-peak strength at a shear displacement of 10 m increased by 3.48% and 3.7%. These phenomena have not been reported in the literature on the strength characteristics of the GMB-S/NW GTX interface, which fully indicates the critical importance of high normal stresses and sufficient shear displacement in revealing the post-shear strength characteristics of the GMB-S/NW GTX interface. 

**Figure 8:**
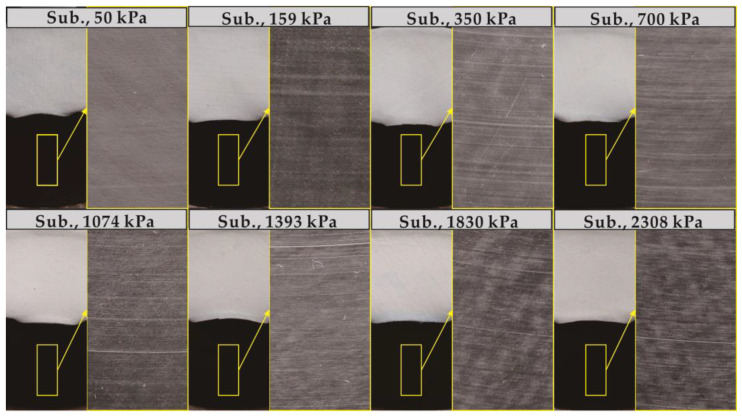
Images of the GMB-S and NW GTX specimens after the ring shear experiment conducted under submerged ambient temperature conditions.

Based on the analysis of Figure 3c,d (plotted using open symbols), the submerged condition had a counteracting effect on the decrease in post-peak at the GMB-S/NW GTX interface compared to the dry condition. The shear mechanism at the GMB-S/NW GTX interface under submerged conditions was inherently different from that under dry conditions. The thickness of the NW GTX specimen under the maximum normal stress of 2308 kPa was 0.26 mm in this experiment, as shown in Figure 5, which was significantly greater than the nail height of the nail plate (0.02 mm) used for clamping the NW GTX specimen in the ring shear experiment. Therefore, the phenomenon of the GMB-S/NW GTX interface post-peak strength decreasing to a minimum value and then increasing again, induced by nail penetration through the NW GTX specimen, should be ruled out. The images of the GMB-S and NW GTX specimens after the ring shear experiment conducted under submerged conditions at ambient temperature are shown in Figure 8. The findings suggest that the annular scratches appeared at a lower normal stress of 159 kPa under submerged conditions than at 700 kPa observed for dry conditions. Additionally, the scratches became more pronounced with increasing normal stress, consistent with the results obtained under dry conditions. Notably, a comparison of Figure 4 and Figure 8 revealed that the GMB-S specimen under submerged conditions exhibited white spots and a noticeably rough texture when touched by hand, indicating that the surface material had been abraded. In contrast, the surface of the GMB-S specimen under dry conditions remained smooth, without a rough surface. These results indicate that the shear mechanism of the GMB-S/NW GTX interface behaves differently under dry and submerged conditions, which is attributed to the variation in surface properties of the GMB-S material caused by material abrasion.

**Figure 9:**
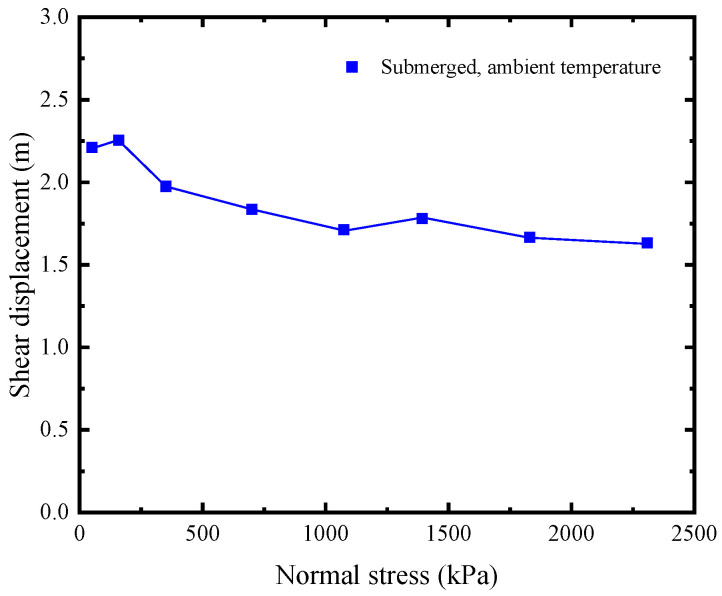
Variations in the shear displacement with normal stress for the GMB-S/NW GTX interface to reach residual strength under submerged conditions.

As shown in Figure 3c,d (plotted using open symbols), the post-peak strength of the GMB-S/NW GTX interface under submerged and high normal stress conditions undergoes a process of initially reaching a minimum value and then slowly increasing with increasing shear displacement. However, it is safer from an engineering design perspective to characterize residual strength using the measured minimum post-peak strength. Figure 9 illustrates the variation in the shear displacement required to achieve the residual strength of the GMB-S/NW GTX interface under different normal stresses. The maximum shear displacement needed to achieve the residual strength of the GMB-S/NW GTX interface is approximately 2.25 m under submerged conditions. Furthermore, the shear displacement required to obtain residual strength generally decreases with increasing normal stress. 

**Figure 10:**
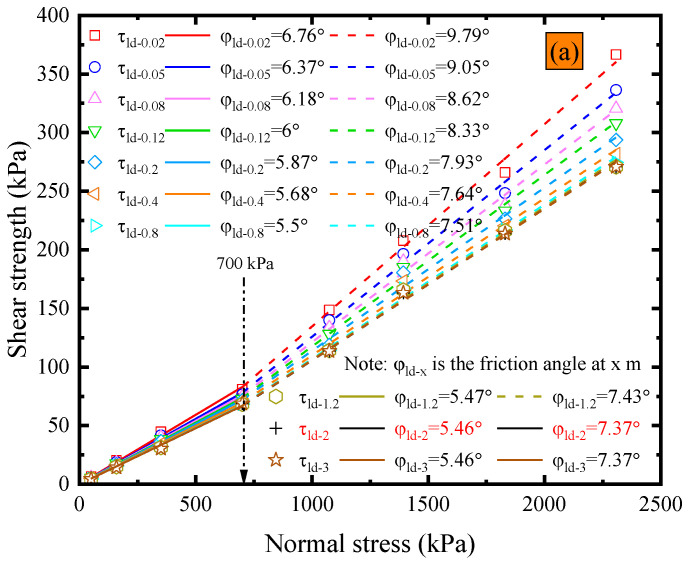

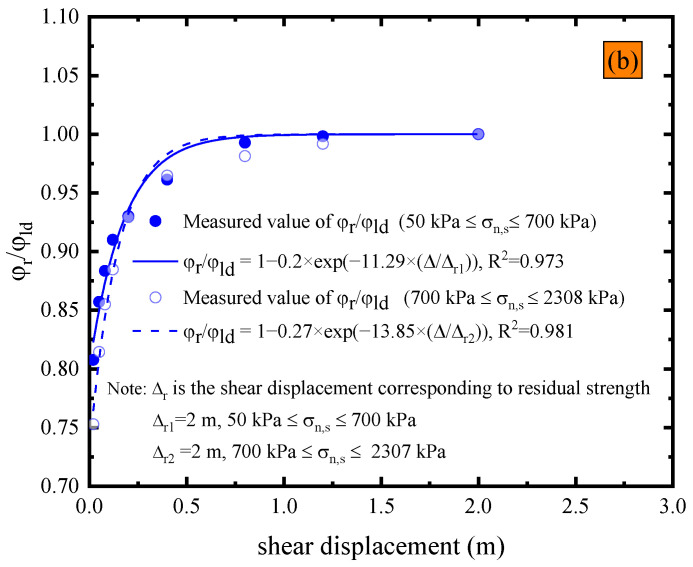
Results for submerged conditions at ambient temperature: (**a**) variations in post-peak strength envelope with shear displacement; (**b**) variations in the ratio of residual friction angle to post-peak large displacement strength friction angle vs. shear displacement.

The post-peak strength envelope with respect to shear displacement is depicted in Figure 10a. It is important to note that the post-peak friction angle at a shear displacement of 3 m is computed based on residual strength data, as the GMB-S/NW GTX interface has already reached residual strength at a shear displacement of 3 m within the normal stress range from 50 kPa to 2308 kPa, as illustrated in Figure 9. As shown in Figure 10a, the residual friction angle at the GMB-S/NW GTX interface exhibits a bilinear characteristic under submerged conditions, with a cross-over point at a normal stress of 700 kPa. This is different from the linear characteristic observed under dry conditions. The residual friction angle corresponding to the normal stress range from 50 kPa to 700 kPa is 5.46°, accounting for only 54.17% of the peak strength. However, the residual friction angle corresponding to the normal stress range from 700 kPa to 2308 kPa is 7.37°, accounting for only 43.12% of the peak strength, as shown in Table 2. Based on the data in Figure 10a, the variations in φ_r_/φ_ld_ vs. shear displacement are shown in Figure 10b. The relationship between the post-peak friction angle and the residual strength friction angle of the GMB-S/NW GTX interface under submerged conditions is also characterized using exponential Equation (2). Therefore, the values of A, B, and Δ_r_ within the normal stress range from 50 kPa to 700 kPa are 0.2, −11.29, and 2, respectively, as given by Equation (3). The values of A, B, and Δ_r_ within the normal stress range from 700 kPa to 2308 kPa are 0.27, −13.85, and 2, respectively, as given by Equation (4). Furthermore, the values of A, B, and Δ_r_ mentioned above are compiled in Table 3.

(3) $$\varphi_{r}=\left(1-0.2\times \exp\left(-11.29\times \left(\frac{\Delta_{\mathrm{ld}}}{2}\right)\right)\right)\times \varphi_{\mathrm{ld}}$$

(4) $$\varphi_{r}=\left(1-0.27\times \exp\left(-13.85\times \left(\frac{\Delta_{\mathrm{ld}}}{2}\right)\right)\right)\times \varphi_{\mathrm{ld}}$$

Lin et al. investigated the strength characteristics of the GMB-S/NW GTX interface under submerged conditions using a direct shear apparatus, with the maximum normal stress reaching 400 kPa [41]. By substituting the experimentally obtained values of peak strength shear displacement (δ_p_), friction angle of large displacement strength (φ_ld_), and shear displacement of large displacement strength (δ_ld_) into Equation (3), the friction angle of peak strength (φ_p_) is calculated to be 14.33°, which is only 5.1% different from the experimental result of 15.1°. Therefore, Equation (3) can be used to reliably evaluate the residual friction angle of the GMB-S/NW GTX interface using the post-peak large displacement friction angle. However, unfortunately, the highest normal stress recorded in historical studies was only 480 kPa, and thus there is a lack of suitable data to further verify the reliability of Equation (4).

In conclusion, the shear displacement required to determine the residual friction angle of the GMB-S/NW GTX interface under submerged conditions should be no less than 2 m. However, as mentioned in Section 1, the direct shear apparatus is limited in handling large shear displacements, which makes it difficult to accomplish this study. Equations (3) and (4) provide a means to evaluate the residual friction angle using the post-peak friction angle, which offers guidance for determining the residual friction angle of the GMB-S/NW GTX interface using direct shear experiments. This is also one of the main objectives of this research. It should be stated that the focus of this study is on the GMB-S/NW GTX interface; therefore, parameters A and B in Equations (2)–(4) are specific to the GMB-S/NW GTX interface only. Parameters corresponding to other types of geomembranes, such as rough geomembranes, will be published in the future.

## 4. Conclusions

A series of direct shear experiments, with a maximum shear displacement of 40 mm, and ring shear experiments, with a shear displacement of 10 m, were performed to investigate the effects of high normal stress (ranging from 50 kPa to 2308 kPa) and specimen conditions (dry and submerged) on the interface shear strength characteristics of the GMB-S/NW GTX interface. Based on the experimental results, the following conclusions were drawn.

(1) The errors of the ring shear and direct shear experiment results (peak friction angle and post-peak friction angle at 40 mm) based on the GMB-S/NW GTX interface were both less than 0.56°, confirming the applicability of the novel ring shear apparatus for studying the strength characteristics of the GMB-S/NW GTX interface. The peak strength of the GMB-S/NW GTX interface exhibited clear bilinear behavior within a normal range from 50 kPa to 2308 kPa, as well as under both dry and submerged conditions. The cross-over point was observed at 1074 kPa, which was one of the selected normal stresses in this experiment. The peak friction angle of the GMB-S/NW GTX interface was slightly smaller under submerged conditions compared to dry conditions under normal stresses ranging from 50 kPa to 1074 kPa. However, the effect of submerged conditions on the peak friction angle was minimal under normal stresses ranging from 1074 kPa to 2308 kPa.
(2) The peak strength of the GMB-S/NW GTX interface exhibited a decreasing trend in shear stress within 10 m under dry conditions at ambient temperature, while it could reach equilibrium within 10 m under submerged conditions, and even the interface shear stress underwent a process of first reaching a minimum value and then increasing again under submerged conditions and high normal stresses under high normal stresses (1830 kPa and 2308 kPa). This could be attributed to greater wear on the surface of the GMB-S specimen under submerged conditions compared to dry conditions.
(3) The residual strength of the GMB-S/NW GTX interface under submerged conditions showed a linear characteristic in the normal stress range from 50 kPa to 2308 kPa. However, the residual friction angle of the interface under submerged conditions exhibited a clear bilinear characteristic, with a cross-over point at 700 kPa. Furthermore, the residual strength was obtained within a depth of 2.25 m under submerged conditions. In addition, it would be relatively safer to select the residual strength under dry conditions at ambient temperature for slope design of landfills with GMB-S and NW GTX materials as liner systems.
(4) This paper revealed the development of the GMB-S/NW GTX interfacial strength within a shear displacement of 10 m under dry and submerged conditions, as well as the method used to determine its residual strength. Three exponential equations were proposed to describe the relationship between the residual friction angle and the post-peak strength friction angle of the GMB-S/NW GTX interface. This can assist a relevant apparatus (i.e., an apparatus with deficiencies in executing large shear displacement) in determining the residual friction angle of the GMB-S/NW GTX interface.

## Figures and Tables

**Table 1:** Summary of the experimental program.

Apparatus	Interface	Specimen Size (mm)	σ_n,s_ ^b^ (kPa)	R ^c^ (mm/min)	Δ ^d^ (mm)	Specimen Interface	Temperature (°C)
DSA ^a^	GMB-S/NW GTX	300 × 300	50, 159, 350, 700, 1074, 1393, 1830, 2308	5	40	Dry	Ambient temperature (16.5–18.3)
RSA ^a^		Φ300/Φ500			10,000	Dry, Submerged	Ambient temperature (16.5–18.3),
							50,
							70

^a^ DSA and RSA are the direct shear apparatus and ring shear apparatus, respectively. ^b^ σ_n,s_ is the normal stress. ^c^ R is the shear rate. ^d^ Δ is the shear displacement.

**Table 2:** Summary of experimental results in friction angles.

Specimen State	Apparatus	Type of Friction Angle	Range of Normal Stress (kPa)	Friction Angle, φ (°) ^b^	R-Squared	Range of Normal Stress (kPa)	Friction Angle, φ (°) ^b^	R-Squared
Dry	DSA ^a^	Peak	50 to 1074	11.55	0.997	1074 to 2308	17.11	0.998
	RSA ^a^			12.11	0.972		16.87	0.998
	DSA ^a^	Post-peak at 40 mm	50 to 2308	8.95	0.993	/	/	/
	RSA ^a^			9.06	0.998			
		Residual		5.23	0.998			
Submerged	RSA ^a^	Peak	50 to 1074	10.08	0.997	1074 to 2308	17.09	0.998
		Residual	50 to 700	5.46	0.997	700 to 2308	7.37	0.999

^a^ DSA and RSA are the direct shear apparatus and ring shear apparatus, respectively. ^b^ φ is the friction angle.

**Table 3:** Summary of A, B, and Δ_r_ in the shear experiment in the GMB-S/NW GTX interface.

Specimen State	Range of Normal Stress (kPa)	A ^a^	B ^a^	Δ_r_^b^ (m)	R-Squared	Range of Normal Stress (kPa)	A ^a^	B ^a^	Δ_r_ ^b^ (m)	R-Squared
Dry	50 to 2308	0.41	−4.23	10	0.976	/	/	/	/	/
Submerged	50 to 700	0.2	−11.29	2	0.973	700 to 2308	0.27	-13.85	2	0.981

^a^ A and B are the coefficients used in Equation (2), respectively. ^b^ Δ_r_ is the shear displacement required to obtain the residual friction angle.

## Data Availability

Not applicable.

## References

[1] Jones D.R.V., Dixon N. (2005). Landfill lining stability and integrity: The role of waste settlement. Geotext. Geomenbrane.

[2] Seo M.W., Park J.B., Park I.J. (2007). Evaluation of interface shear strength between geosynthetics under wet condition. Soils Found..

[3] Kim D., Frost J.D. (2011). Effect of geotextile constraint on geotextile/geomembrane interface shear behavior. Geosynth. Int..

[4] Bacas B.M., Cañizal J., Konietzky H. (2015). Shear strength behavior of geotextile/geomembrane interfaces. J. Rock Mech. Geothch..

[5] Cen W.J., Wang H., Sun Y.J. (2018). Laboratory investigation of shear behavior of high-density polyethylene geomembrane interfaces. Polymers.

[6] Chang J.Y., Feng S.J. (2021). Dynamic shear behaviors of textured geomembrane/nonwoven geotextile interface under cyclic loading. Geotext. Geomenbrane.

[7] Karademir T., Frost J.D. (2011). Elevated Temperature Effects on Geotextile-Geomembrane Interfacial strength. Geo-Frontiers Congress.

[8] Fan J., Rowe R.K. (2022). Piping of silty sand tailings through a circular geomembrane hole. Geotext. Geomenbrane.

[9] Qian X.D., Koerner R.M., Gray D.H. (2002). Geotechnical Aspect of Landfill Design and Construction.

[10] Mitchell J.K., Seed R.B., Seed H.B. (1990). Kettleman Hills waste landfill slope failure. I: Liner-system properties. J. Geotech. Eng..

[11] Seed R.B., Mitchell J.K., Seed H.B. (1990). Kettleman Hills waste landfill slope failure. II: Stability analyses. J. Geotech. Eng..

[12] Koerner R.M. (2012). Designing with Geosynthetics.

[13] Stark T.D., Poeppel A.R. (1994). Landfill liner interfacial strengths from torsional-ring-shear tests. J. Geotech. Eng..

[14] Blight G. (2008). Slope failures in municipal solid waste dumps and landfills: A review. Waste Manag. Res..

[15] Jahanfar A., Gharabaghi B., Mcbean E.A., Dubey B.K. (2017). Municipal solid waste slope stability modeling: A probabilistic approach. J. Geotech. Geoenviron..

[16] Kocasoy G., Curi K. (1995). The umraniye-hekimbasi open dump accident. Waste Manag. Res..

[17] Eid H.T., Stark T.D., Evans W.D., Sherry P.E. (2000). Municipal solid waste slope failure. i: Waste and foundation soil properties. J Geotech. Geoenviron..

[18] Caicedo B., Yamin L., Giraldo E., Coronado O., Soler N. (2002). Geomechanical properties of municipal solid waste in Dona Juana sanitary landfill. Proceedings of the Fourth International Congress on Environmental Geotechnics.

[19] Huvaj-Sarihan N., Stark T.D. Back analyses of landfill slope failures. Proceedings of the 6th International Conference on Case Histories in Geotechnical Engineering and Symposium in Honor of Professor James K. Mitchell.

[20] Lavigne F., Wassmer P., Gomez C., Davies T.A., Sri Hadmoko D., Iskandarsyah T.Y.W., Pratomo I. (2014). The 21 February 2005, catastrophic waste avalanche at Leuwigajah dumpsite, Bandung, Indonesia. Geoenviron. Disasters.

[21] Koelsch F., Fricke K., Mahler C., Damanhuri E. Stability of landfills-The Bandung dumpsite disaster. Proceedings of the 10th International Waste Management and Landfill Symposium.

[22] Zhan L.T., Guan R.Q., Chen Y.M., Liu Z. (2010). Monitoring and back analyses of slope failure process at a landfill. Chin. J. Rock Mech. Eng..

[23] Athanasopoulos G., Vlachakis V., Zekkos D., Spiliotopoulos G. The December 29th 2010 Xerolakka municipal solid waste landfill failure. Proceedings of the 18th International Conference on Soil Mechanics and Geotechnical Engineering.

[24] Zhan T.L., Zhan X., Lin W., Luo X., Chen Y. (2014). Field and laboratory investigation on geotechnical properties of sewage sludge disposed in a pit at Changan landfill, Chengdu, China. Eng. Geol..

[25] Du Y., Fu H., Liu L., Feng G., Wen D., Peng X., Ding H. (2021). Continued Monitoring and Modeling of Xingfeng Solid Waste Landfill Settlement, China, Based on Multiplatform SAR Images. Remote Sens..

[26] Feng S.J., Chang J.Y., Zhang X.L., Shi H., Wu S.J. (2021). Stability analysis and control measures of a sanitary landfill with high leachate level. J. Geotech. Geoenviron..

[27] Krushelnitzky R.P., Brachman R.W.I. (2009). Measured deformations and calculated stresses of high-density polyethylene pipes under very deep burial. Can. Geotech. J..

[28] Lupo J.F. (2010). Liner system design for heap leach pads. Geotext. Geomenbranes.

[29] Seeger S., Böhm H., Söhring G., Müller W. Long term testing of geomembranes and geotextiles under shear stress. Proceedings of the Second European Geosynthetics Conference, Pàtron Editore.

[30] Stark T.D., Choi H. (2004). Peak vs. residual interfacial strengths for landfill liner and cover design. Geosynth. Int..

[31] Seed R.B., Boulanger R.W. (1991). Smooth HDPE-clay liner interface shear strengths: Compaction effects. J. Geotech. Eng..

[32] Byrne R.J., Kendall J., Brown S. Cause and mechanism of failure Kettleman Hills landfill B-19, phase IA. Proceedings of the ASCE Specialty Conference on Stability and Performance of Slope and Embankments-II.

[33] Stark T.D. (1999). Stability of waste containment facilities. Proceedings of Waste Tech 99.

[34] Rowe R.K., Yu Y. (2019). Magnitude and significance of tensile strains in geomembrane landfill liners. Geotext. Geomenbranes.

[35] Liu Z.L., Shi J., Zhang Y.C., Xu G.J., Shu S., Li Y.P., Lei G.H. (2022). A Novel Multifunctional Ring Shear Apparatus for Investigating the Interfacial strength Characteristics of Liner Systems. Geotech. Test. J..

[36] Jones D.R.V., Dixon N. (1998). Shear strength properties of geomembrane/geotextile interfaces. Geotext. Geomenbrane.

[37] Frost J.D., Lee S.W. (2001). Microscale study of geomembrane-geotextile interactions. Geosynth. Int..

[38] Wasti Y., Özdüzgün Z.B. (2001). Geomembrane–geotextile interface shear properties as determined by inclined board and direct shear box tests. Geotext. Geomenbranes.

[39] Akpinar M.V., Benson C.H. (2005). Effect of temperature on shear strength of two geomembrane-geotextile interfaces. Geotext. Geomenbrane.

[40] Bergado D.T., Ramana G.V., Sia H.I. (2006). Evaluation of interface shear strength of composite liner system and stability analysis for a landfill lining system in Thailand. Geotext. Geomenbrane.

[41] Lin H., Han Z.W., Shi J. (2020). Interface shear failure mechanisms and peak strength ananlysis of geosynthetics. J. Huazhong Univ. Sci. Technol. (Nat. Sci. Ed.).

[42] Vaid Y.P. (1995). Geomembrane Coefficients of Interface Friction. Geosynth. Int..

[43] Stark T.D., Eid H.T. (1996). Shear Behavior of Reinforced Geosynthetic Clay Liners. Geosynth. Int..

[44] Tan S.A., Chew S.H., Wong W.K. (1998). Sand–geotextile interface shear strength by torsional ring shear tests. Geotext. Geomenbranes.

[45] Fleming I.R., Sharma J.S., Jogi M.B. (2006). Shear strength of geomembrane–soil interface under unsaturated conditions. Geotext. Geomenbranes.

[46] Eid H.T. (2011). Shear Strength of Geosynthetic Composite Systems for Design of Landfill Liner and Cover Slopes. Geotext. Geomenbranes.

[47] Negussey D., Wijewickreme W.K.D., Vaid Y.P. (1989). Geomembrane Interface Friction. Can. Geotech. J..

[48] Lin H., Shi J., Qian X., Zhang L. (2014). An improved simple shear apparatus for GCL internal and interface stress-displacement measurements. Environ. Earth Sci..

[49] (2012). Standard Test Method for Measuring the Nominal Thickness of Geosynthetics.

[50] (2010). Standard Test Method for Measuring Mass Per Unit Area of Geotextiles.

[51] (2021). Standard Test Method for Determining the Shear Strength of Soil-Geosynthetic and Geosynthetic-Geosynthetic Interfaces by Direct Shear.

[52] (2020). Standard Test Method for Determining the Internal and Interface Shear Resistance of Geosynthetic Clay Liner by the Direct Shear Method.

[53] Feng S.J., Cheng D. (2014). Shear strength between soil/geomembrane and geotextile/geomembrane interfaces. Tunneling and Underground Construction GSP 242.

[54] Feng S.J., Lu S.F. (2016). Repeated shear behaviors of geotextile/geomembrane and geomembrane/clay interfaces. Environ. Earth Sci..

[55] Abdelaal F.B., Solanki R. (2022). Effect of geotextile ageing and geomembrane surface roughness on the geomembrane-geotextile interfaces for heap leaching applications. Geotext. Geomenbrane.

[56] Chang M. (2005). Three-dimensional stability analysis of the Kettleman Hills landfill slope failure based on observed sliding-block mechanism. Comput. Geotech..

[57] Zhang Z., Wang Y., Fang Y., Pan X., Zhang J., Xu H. (2020). Global study on slope instability modes based on 62 municipal solid waste landfills. Waste Manag. Res..

[58] Stark T.D., Williamson T.A., Eid H.T. (1996). HDPE geomembrane/geotextile interface shear strength. J. Geotech. Geoenviron..

[59] Kim D. (2006). Multi-Scale Assessment of Geotextilegeomembrane Interaction. Ph.D. Thesis.

[60] Kwak C.W., Park I.J., Park J.B. (2013). Modified cyclic shear test for evaluating disturbance function and numerical formulation of geosynthetic-soil interface considering chemical effect. Geotech. Test. J..

